# Effect of Prior Atorvastatin Treatment on the Frequency of Hospital Acquired Pneumonia and Evolution of Biomarkers in Patients with Acute Ischemic Stroke: A Multicenter Prospective Study

**DOI:** 10.1155/2017/5642704

**Published:** 2017-03-05

**Authors:** Yuetian Yu, Cheng Zhu, Chunyan Liu, Yuan Gao

**Affiliations:** ^1^Department of Critical Care Medicine, Renji Hospital, School of Medicine, Shanghai Jiao Tong University, Shanghai 200001, China; ^2^Department of Emergency, Ruijin Hospital, School of Medicine, Shanghai Jiao Tong University, Shanghai 200025, China; ^3^Department of Emergency, Minhang District Central Hospital, Shanghai 201100, China

## Abstract

*Objective*. To investigate whether prior treatment of atorvastatin reduces the frequency of hospital acquired pneumonia (HAP).* Methods*. Totally, 492 patients with acute ischemic stroke and Glasgow Coma Scale ≤ 8 were enrolled in this study. Subjects were assigned to prior atorvastatin treatment group (*n* = 268, PG) and no prior treatment group (*n* = 224, NG). All the patients were given 20 mg atorvastatin every night during their hospital stay. HAP frequency and 28-day mortality were measured. Levels of inflammatory biomarkers [white blood cell (WBC), procalcitonin (PCT), tumor necrosis factor-alpha (TNF-*α*), and interleukin-6 (IL-6)] were tested.* Results*. There was no significant difference in the incidence of HAP between PG and NG (25.74% versus. 24.55%, *p* > 0.05) and 28-day mortality (50.72% versus 58.18%, *p* > 0.05). However, prior statin treatment did modify the mortality of ventilator associated pneumonia (VAP) (36.54% versus 58.14%, *p* = 0.041) and proved to be a protective factor (HR, 0.564; 95% CI, 0.310~0.825, *p* = 0.038). Concentrations of TNF-*α* and IL-6 in PG VAP cases were lower than those in NG VAP cases (*p* < 0.01).* Conclusions*. Prior atorvastatin treatment in patients with ischemic stroke was associated with a lower concentration of IL-6 and TNF-*α* and improved the outcome of VAP. This clinical study has been registered with ChiCTR-ROC-17010633 in Chinese Clinical Trial Registry.

## 1. Background

Hospital acquired pneumonia (HAP) which constitutes a frequent infection in intensive care unit (ICU) patients consumes vast healthcare resources and increases proportionally to the duration of ICU stay. Ventilator associated pneumonia (VAP) is defined as HAP in patients receiving mechanical ventilation [1, 2]. The incidence of HAP depends on the population studied. For example, in patients with ischemic stroke who are characterized by advanced age, depressed level of consciousness, immune suppression, and long-term bed rest, the incidence of HAP can increase to approximately 40%. It is recognized that one-third to half of all HAP-related deaths are directly attributable to pneumonia [3]. Despite improvements in bundle care prevention, mortality continues to remain high. Therefore, new effective adjunctive therapies are still needed for HAP prevention and treatment.

Statins (3-hydroxy-3-methylglutaryl coenzyme A reductase inhibitors) present anti-inflammatory effects besides their ability to regulate cholesterol composition these years [4, 5]. Studies have also found statins to possess immunomodulating properties and to be linked to favorable outcomes from sepsis [6].

However, little is known about the mechanisms by which statins counteract inflammation and there is still uncertainty about the risks and benefits of administering statins de novo or in continuing statin therapy in patients with infectious diseases [7–9]. It has therefore been deduced that these pleiotropic characteristics of statins might relate to cumulative statins treatment time and their concentration in blood. We hypothesized that statins treatment for a long time might decrease the incidence of HAP and influence the inflammatory response to infection.

## 2. Materials and Methods

The present study is a three-center, two-group, prospective observational cohort trial. All the three teaching hospitals are affiliated to Shanghai Jiaotong University School of Medicine (155 ICU beds in total). The study was approved by the Review Board and Ethics Committee of Shanghai Jiaotong University and informed consent was obtained for all patients, from either the patient or the next of kin. The study took place at the three ICUs from January 2008 to December 2013.

### 2.1. Study Population

Consecutive sampling was used to recruit critical patients. Patients were eligible for enrolment if they were diagnosed with acute ischemic stroke by magnetic resonance imaging (MRI); were aged between 18 and 80 years; had Glasgow Coma Scale (GCS) ≤ 8. Patients were excluded from the study if they were moribund or not expected to survive 28 days because of an underlying irreversible medical condition; had active pneumonia when admitted to ICU or had extrapulmonary infection during ICU stay; had a known intolerance to statins or were unable to have enteral administration of statin; had severe liver disease (acute liver failure or chronic liver disease with Child-Pugh classification C); had a serum alanine aminotransferase (ALT) or aspartate aminotransferase (AST) level greater than three times the normal value or a serum creatinine kinase (CK) level greater than five times the upper limit of normal levels; had commenced statin therapy less than 4 weeks before hospital admission or had statin stopped for more than 72 hours before the development of ischemic stroke; were pregnant or if informed consent could not be obtained.

### 2.2. Study Protocol

All the patients eligible were enrolled in this research during our study period. Depending on previous statin administration, they were classified into prior atorvastatin treatment group (prior use atorvastatin for more than one month before being admitted to ICU, PG) and no prior treatment group (did not use atorvastatin before being admitted to ICU, NG).

All the patients were followed up and were divided by the Oxfordshire Community Stroke Project (OCSP) into four subtypes as total anterior circulation infarcts (TACI), partial anterior circulation infarcts (PACI), posterior circulation infarcts (POCI), and lacunar infarcts (LACI) [10]. All the patients selected were cared for in the ICUs by doctors on duty. Therapeutic schedule was established during ward rounds (three times daily at 8:00, 15:00, and 22:00) by attending doctors or at any emergency moment of the patients. In accordance with the guidelines by the American Heart Association and American Stroke Association [11, 12] and the secondary prevention strategy after ischemic stroke [13, 14], all the patients were ordered atorvastatin which is the most commonly prescribed statin in China. Atorvastatin 20 mg was administered every night via enteral feeding tube or per os during stay in ICU for up to 28 days or until death or discharge from the ICU, whichever of these occurred first [15]. A 20 mg dose was chosen (lower end of the dosing range) because of current safety concerns and previous pharmacokinetic study suggesting high plasma levels upon administration [16].

### 2.3. Clinical Assessment

Baseline assessment included the evaluation of demographic data (age and gender), medical history, stroke subtype, need for invasive positive pressure ventilation (IPPV), ratio of partial oxygen to fraction of inspired oxygen (PaO_2_/FiO_2_), acute physiology and chronic health evaluation score (APACHE II), Glasgow Coma Scale (GCS), modified clinical pulmonary infection score (CPIS), lipid concentration, hepatic function, and level of inflammatory biomarkers.

### 2.4. Diagnosis, Treatment, and Prevention of HAP or VAP

HAP is suspected if the patient has a radiographic infiltrate that is new or progressive, along with clinical findings that suggest infection, which include the onset of fever (temperature ≥ 38.3°C), leukocytosis (≥10 × 10^9^/L or ≤4 × 10^9^/L), purulent sputum, and decline in oxygenation. In addition, a positive tracheal aspirate quantitative culture (≥10^5^ colony-forming units/mL) or a positive bronchoalveolar lavage fluid (BALF) culture (≥10^4^ colony-forming units/mL) is required to confirm the diagnosis of HAP. When this definition has been applied to mechanically ventilated patients, then VAP is suspected [17, 18].

The protocol for HAP or VAP treatment and prevention followed standard protocols in all institutions also based on accepted guidelines. Care bundles for preventing HAP or VAP were also used including daily assessments of sedation, daily oral hygiene, the use of a semirecumbent position with the head of the bed elevated to at least 30°, peptic ulcer prophylaxis, and venous thromboembolism prophylaxis [17–19].

One month before the start of this study, a standardized sampling, processing, analysis, and statistics procedure was set by 6 investigators from these centers after 3 days' learning and discussion. All of the centers carefully followed this procedure during the study time. There was no significant difference in compliance among the three centers.

### 2.5. Measurement of Blood and Microbiology

Blood samples were collected for the measurement of inflammatory biomarkers at baseline and at the first, third, fifth, seventh, and tenth day after the clinical diagnosis of HAP or VAP. ALT, AST, and CK were tested on Monday and Thursday every week. All the blood samples were separated by centrifugation (TDL-60C, 6000 revolutions per minute, China). Commercially available ELISA assays were used for detecting inflammatory biomarkers. White blood cell (WBC) and differential blood counts were determined using a Coulter STKS clinical analyzer (Coulter Electronics Inc., Miami, FL, USA). Procalcitonin (PCT) levels were obtained using the enzyme-linked fluorescent assay (ELFA) technique. This assay combines a one-step immunoassay sandwich method with a final automatic fluorescent detection by a VIDAS instrument (VIDAS BRAHMS PCT, bioMérieux, Lyon, France). The measurement range of this instrument was set to 0.05~200 ng/mL. Serum concentrations of interleukin-6 (IL-6) and tumor necrosis factor-alpha (TNF-*α*) were measured using a CBA kit (BDTM Cytometric Bead Array-Human Inflammatory cytokine kit) The detection limit of the methods for the TNF-*α* was 20 pg/mL and 1.88~120 ng/mL for IL-6.

To determine which bacterial species was responsible for pneumonia, bronchial secretions or BALF were sampled at the baseline and at the first, third, fifth, seventh, and tenth day after clinical diagnosis of HAP or VAP. The diagnostic flexible bronchoscopy guideline of British Thoracic Society [20] was followed. Microbiology assessment included identification and quantitative assessment of the microbial agent and evaluation of its in vitro resistance to antibiotics.

### 2.6. Outcomes

We primarily assessed the morbidity of HAP or VAP and mortality over a 28-day treatment in ICU or at the end of ICU treatment. In addition, we assessed the evolution of inflammatory biomarkers (WBC; PCT; TNF-*α*; IL-6). Adverse events related to atorvastatin treatment (CK abnormal changes, hepatic enzyme dysfunction) were also documented.

### 2.7. Statistical Analysis

Statistical analysis was performed by using SPSS version 19.0 (IBM for Windows). Data were initially assessed for normality and log transformed where appropriate. Data between the PG and NG were compared using Chi-square test for equal proportion or Fisher exact test where numbers were small with results presented as percentages (*n*). Continuously normally distributed variables were compared using Student's *t*-test and presented as means (standard deviations), whereas nonnormally distributed data was compared using Wilcoxon rank-sum test and reported as medians (interquartile range). Differences in changes of inflammatory biomarkers (dependent variables) with time were analyzed by using a general linear model for repeated measures with Bonferroni's adjustment. Logistic regression models were used for multivariate analysis, in which we included all variables found to be related to outcomes in univariate analysis. The probability of ICU survival was assessed with Kaplan-Meier survival analysis, and comparisons between groups were performed using the log-rank (Mantel-Cox) test. Cox proportional hazards regression analysis was also used to examine the effect of multiple risk factors on mortality. Factors associated significantly (*p* < 0.05) with mortality in univariate analyses were entered in multivariable analyses. The statistical tests performed were two-sided. All analysis was performed on an intention-to-treat basis and a two-sided *p* < 0.05 was considered to be statistically significant. Figures were drawn by using GraphPad prism version 5.0 (GraphPad Software for Windows).

## 3. Results

**Figure 1:**
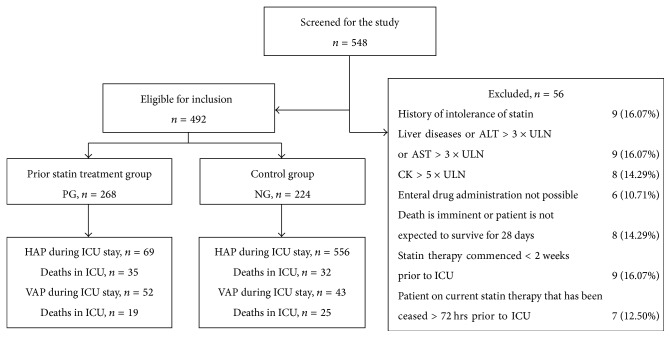
Flowchart of the study. ULN: upper limit of normal.

A total of 548 patients were screened and 492 eligible patients were recruited for the study (102 patients were enrolled in Renji Hospital, 217 patients were enrolled in Ruijin Hospital, and 173 patients were enrolled in Minhang District Central Hospital). A total of 268 patients were assigned to PG, and 224 patients to NG. Fifty-six patients who met the exclusion criteria were excluded from the study (Figure 1).

### 3.1. Baseline

Four hundred and ninety-two eligible patients were recruited in the final analysis. The baseline characteristics of all 492 patients are presented at Table 1. The demographic data, position of stroke, PaO_2_/FiO_2_, APACHE II, GCS, modified CPIS, hepatic function, and levels of inflammatory biomarkers were not significantly different between PG and NG. As expected, patients in PG had significantly higher rates of ischemic heart disease, ischemic cerebral disease, and a lower level of lipid because of long time treatment of statins.

### 3.2. Primary Outcomes

One hundred and twenty-four patients (25.20%) were diagnosed with HAP during the 28-day treatment in ICU. Sixty-nine patients (25.74%) were from PG and 55 (24.55%) from NG. Thirty-five patients (50.72%) in PG and 32 patients (58.18%) in NG died during the 28-day treatment period in ICU. Although there was an indication of a trend toward a higher mortality in NG, there was no statistical significant difference in 28-day ICU mortality or the incidence of HAP between the two groups (*p* > 0.05).

The ICU treatment duration before HAP diagnosis was 13.15 days in PG and 12.68 days in NG. The incidence of septic shock was 30.43% in PG and 34.55% in NG. Body temperature, WBC, PCT, TNF-*α*, and IL-6 were not different between the PG and NG at the day of diagnosed HAP (*p* > 0.05, Table 2).

In the HAP patients, a total of 11 different species of microorganisms were isolated. The most frequently isolated microorganisms were* Pseudomonas aeruginosa* (33.2%) and* Klebsiella* species (28.5%). In 19 patients, more than one pathogen was detected. Univariate analysis revealed no significant differences between the two groups for the types of pathogens.

**Figure 2:**
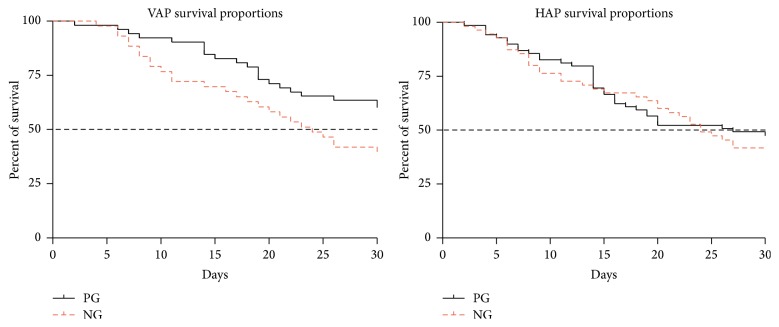
Probability of survival of the patients with HAP or VAP during 28-day ICU treatment. The dashed black line refers to 50% of survival (median survival reference line).

Two hundred and thirty-three (47.35%) patients enrolled in the study needed IPPV therapy for more than 48 h. Ninety-five patients (40.77%) with IPPV therapy presented with VAP during the 28-day treatment period in the ICU. Subtypes of stroke, modified CPIS, GCS, and APACHE II of the patients with VAP were presented in Table 3. The variables were similar (*p* > 0.05). The incidence of VAP was not significantly different between the two groups (39.39% versus 42.57%, *p* = 0.581). However, prior statin therapy did modify the outcome of VAP (36.54% versus 58.14%, *p* = 0.041, Figure 2). Cox regression analysis for the effect of multiple factors on VAP mortality after 28-day ICU treatment is given in Table 4. Prior statin treatment proved to be the one protective factor (HR, 0.564; 95% CI, 0.310~0.825, *p* = 0.038, Table 4).

### 3.3. Secondary Outcomes

**Figure 3:**
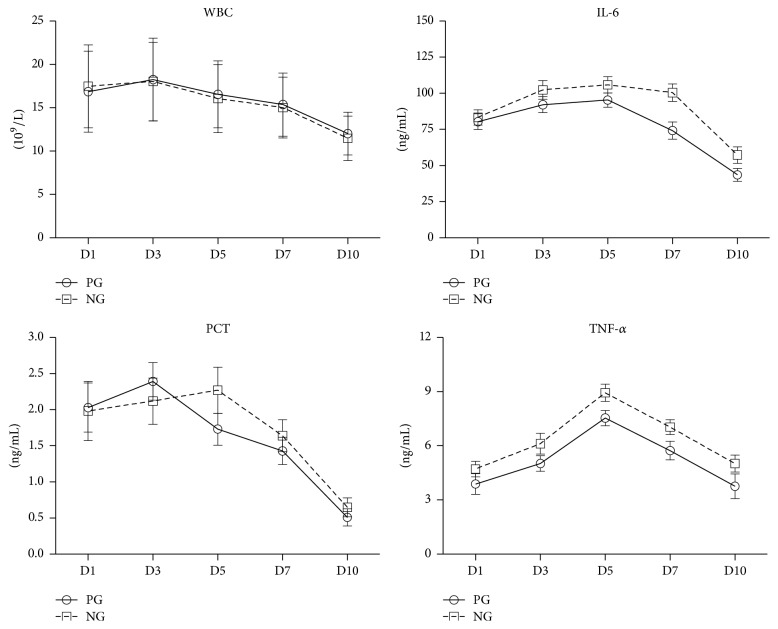
Evolution of WBC, PCT, TNF-*α*, and IL-6 in blood serum in VAP cases.

Inflammatory biomarkers were collected and measured at the first, third, fifth, seventh, and tenth day after the clinical diagnosis of VAP. WBC and PCT assessed during the study were not significantly different between PG and NG in serum (*p* > 0.05). However, the concentrations of TNF-*α* and IL-6 in PG VAP cases were lower than those in NG VAP cases in serum (*p* < 0.01, Table 5, Figure 3).

### 3.4. Adverse Effects

Atorvastatin administered at 20 mg every night was relatively safe in the study group. Serious adverse events as defined herein did not occur in the present study. Slight elevations in CK occurred in both PG and NG. Hepatic function was similar between the two groups. Myopathy was not diagnosed.

## 4. Discussion

### 4.1. Key Findings

In this prospective observational study, we found that therapy with statins for at least one month before the onset of acute ischemic stroke was probably associated with a decreased mortality of VAP during 28-day ICU treatment. Concentrations of TNF-*α* and IL-6 in PG VAP cases were lower than those in NG VAP cases in serum.

### 4.2. Relationship with Previous Studies

The findings of our study suggest that prior statins treatment may be useful as an adjunctive therapy in critically ill patients and notably improves the outcome. In this respect, our results support the findings of previous studies which suggest that statins may favorably affect critically ill patients or animals [21–24]. Although previous studies demonstrated a beneficial effect of prior statin use on the outcome of patients with pneumonia, a large prospective study came to a different conclusion which suggested a potentially harmful risk of statins [9, 25]. In our opinion, it should be pointed out that those previous studies neither classified the patients who had already been on chronic statin treatment nor considered the plasma statins concentration. The subjects of our study were carefully selected to avoid misinterpretation of results.

One previous study provides evidence that the addition of oral pravastatin in the usual treatment regime of ICU patients might have significant effects on mortality of more severely ill patients (APACHE II ≥ 15) [21]. A majority of the patients included in our study were more severe than those in the previous study. The average of APACHE II was higher than 15, which means that the assumed mortality of our patients was higher than 25%. Our data suggested that the probability of survival during the 28-day treatment period was decreased in PG compared to that of NG (*p* = 0.041). These findings suggest that statins may be useful as an adjunctive therapy in critically ill patients. In this respect, our results support the findings of previous studies which suggested that statins might favorably affect the course of critically ill patients [21].

### 4.3. Study Significance

In the present study, we evaluated the effect of prior treatment of atorvastatin on HAP or VAP frequency in ischemic stroke patients. To the best of our knowledge, this is the first study which provides evidence supporting the idea that prior statins treatment might affect the frequency of HAP or VAP in patients with ischemic stroke and decrease the inflammatory response. Our findings suggest that although prior treatment with atorvastatin did not significantly affect the incidence of HAP or VAP, it did modify the outcome of VAP. Additionally, continued use of atorvastatin therapy in prior statin users was associated with lower levels of TNF-*α* and IL-6 in serum. This suggests a potential benefit of continued administration or de novo use in infectious diseases. We assume that this might be explained by potential “anti-inflammatory” or “anti-infectious” capability of statins, which may reduce the inflammatory burden of the most severe forms of infectious illness such as VAP [2, 26].

In this investigation, we mainly followed the evolution of clinical and biological inflammatory biomarkers that are commonly used in ICU and we have not assessed specific pathways that have been reported to be affected by statins. The levels of TNF-*α* and IL-6 were decreased by atorvastatin treatment in PG patients with VAP. It should also be noted here that the properties of statins act through down- or upregulation of cytokines, modification of the function of leukocytes and lymphocytes, and direct inhibition of major histocompatibility complex II [27]. Furthermore, it was previously reported that statins can cause an increase in heme-oxygenase activity and may reduce the oxidative burden in tissues during sepsis [28]. Therefore, statins may modify the inflammatory cascade and inhibit the progression of septic conditions. TNF-*α* and IL-6 are pleiotropic cytokines and central mediators of the acute-phase response. They are produced by vascular endothelial cells, smooth muscle cells, and leukocytes with a broad range of effects on diverse immune cells [29, 30]. Atorvastatin is anti-inflammatory and acts by downregulating cytokines in the endothelium and leukocytes. In this study, atorvastatin decreased the concentrations of TNF-*α* and IL-6 in serum. However, these anti-inflammatory effects may need sustained statin treatment so as to maintain a relatively high plasma concentration of statin.

### 4.4. Study Limitations

We acknowledge that there are limitations to this study. We included a homogeneous population of ischemic stroke which might minimize the heterogeneity and avoided misinterpreting the study result. Atorvastatin was the only type of statins in our study and no placebo arm was established. We also could not provide the factors associated with VAP mortality. So the application to routine clinical practice of other statins (simvastatin, pravastatin, rosuvastatin, etc.) remains uncertain. Whether the prior treatment of atorvastatin in general may translate into clinical benefits outside this setting still deserves further explorations.

## 5. Conclusion

In conclusion, the present investigation is the first prospective study that assessed the impact of statins in patients with ischemic stroke in their usual treatment. Our findings suggest that prior atorvastatin treatment in ICU may act to decrease the concentrations of TNF-*α* and IL-6. In addition, it may have a beneficial role in the outcome of VAP.

## Figures and Tables

**Table 1:** Baseline characteristics of all 492 patients.

Variable	PG (*n* = 268)	NG (*n* = 224)	*p*
Male gender, *n* (%)	130 (48.51)	108 (48.21)	1.000
Age, yrs	68.34 ± 7.73	67.13 ± 7.82	0.085
Previous medical illness			
Cardiovascular disease, *n* (%)	175 (65.30)	123 (54.91)	0.019
Cerebrovascular disease, *n* (%)	110 (41.04)	70 (31.25)	0.025
Position of stroke			
TACI, *n* (%)	64 (23.88)	49 (21.88)	0.838
PACI, *n* (%)	51 (19.03)	46 (20.54)	
POCI, *n* (%)	68 (25.37)	52 (23.21)	
LACI, *n* (%)	85 (31.72)	77 (34.37)	
IPPV, *n* (%)	132 (49.25)	101 (49.05)	0.366
Modified CPIS	3.35 ± 1.22	3.58 ± 1.72	0.084
GCS	5.32 ± 1.34	5.08 ± 1.74	0.085
APACHE II	17.56 ± 3.43	18.24 ± 4.32	0.052
PaO_2_/FiO_2_ (mmHg)	332.45 ± 43.55	340.47 ± 48.58	0.054
Cholesterol (mmol/L)	4.58 ± 0.76	5.13 ± 1.10	0.001
LDL (mmol/L)	3.13 ± 0.45	3.19 ± 0.34	0.001
ALT (U/L)	58.33 ± 5.45	57.65 ± 5.52	0.170
AST (U/L)	23.09 ± 3.15	23.57 ± 2.45	0.064
CK (ng/mL)	45.33 ± 3.56	46.01 ± 4.89	0.076
WBC (10^9^/L)	8.72 ± 1.43	8.55 ± 0.93	0.127
PCT (ng/mL)	0.03 ± 0.01	0.03 ± 0.01	1.000
TNF-*α* (ng/L)	0.55 ± 0.09	0.56 ± 0.08	0.198
IL-6 (ng/mL)	28.54 ± 5.87	27.67 ± 5.98	0.105

**Table 2:** Characteristics at the day of HAP diagnosis.

Variable	PG (*n* = 69)	NG (*n* = 55)	*p*
MDR cases, *n* (%)	32 (46.38)	27 (49.09)	0.857
Septic shock, *n* (%)	21 (30.43)	19 (34.55)	0.700
ICU stay before HAP, (days)	13.15 ± 4.32	12.68 ± 5.01	0.576
Temperature (°C)	38.03 ± 0.45	38.18 ± 0.53	0.091
WBC (10^9^/L)	15.73 ± 3.67	16.04 ± 3.05	0.616
PCT (ng/mL)	1.34 ± 0.23	1.25 ± 0.32	0.071
TNF-*α* (ng/L)	2.87 ± 0.34	2.76 ± 0.46	0.129
IL-6 (ng/mL)	85.56 ± 7.78	83.57 ± 6.87	0.139

**Table 3:** Characteristics of IPPV patients at the day of VAP diagnosis.

Variable	PG (*n* = 52)	NG (*n* = 43)	*p*
Position of stroke			
TACI, *n* (%)	9 (17.31)	6 (13.95)	0.780
POCI, *n* (%)	43 (82.69)	37 (86.05)	
Modified CPIS	8.11 ± 2.75	8.23 ± 2.56	0.828
GCS	4.12 ± 0.87	4.02 ± 0.73	0.551
APACHE II	22.05 ± 3.39	21.87 ± 3.47	0.799

**Table 4:** Cox regression analysis for the effect of multiple factors on mortality of patients with HAP.

Variable	HR	95% CI	*p*
Prior statin treatment	0.564	0.310~0.825	0.038
Age	2.683	1.304~4.668	0.041
Gender (male)	1.083	0.835~1.476	0.882
Position of stroke	4.494	1.086~9.598	0.032
CPIS	3.884	2.883~7.936	0.042
GCS	2.674	1.983~8.836	0.036
APACHE II	2.563	1.376~8.763	0.042
Cholesterol	1.542	0.732~6.424	0.453
LDL	1.145	0.832~5.324	0.464
Previous cardiovascular disease	1.185	0.860~4.562	0.645
Previous cerebrovascular disease	1.874	0.874~7.5124	0.644

**Table 5:** Inflammatory markers at the day of VAP diagnosis and 10 days of treatment.

Variable	PG	NG	*p*
Day of VAP diagnosis	*n* = 52	*n* = 43	
WBC (10^9^/L)	16.85 ± 4.65	17.46 ± 4.78	0.531
PCT (ng/mL)	2.03 ± 0.34	1.98 ± 0.41	0.517
TNF-*α* (ng/L)	3.88 ± 0.57	4.71 ± 0.42	0.001
IL-6 (ng/mL)	80.34 ± 5.45	83.25 ± 5.28	0.010
10 days of treatment	*n* = 49	*n* = 38	
WBC (10^9^/L)	12.02 ± 2.45	11.47 ± 2.56	0.311
PCT (ng/mL)	0.51 ± 0.12	0.53 ± 0.13	0.459
TNF-*α* (ng/L)	3.76 ± 0.68	5.02 ± 0.47	0.001
IL-6 (ng/mL)	43.54 ± 4.45	57.25 ± 5.68	0.001

## Abbreviations

ALT: Alanine aminotransferase
APACHE II: Acute physiology and chronic health evaluation II
AST: Aspartate aminotransferase
BALF: Bronchoalveolar lavage fluid
CK: Creatinine kinase
CPIS: Clinical pulmonary infection score
ELISA: Enzyme-Linked Immunosorbent Assay
GCS: Glasgow Coma Scale
HAP: Hospital acquired pneumonia
ICU: Intensive care unit
IL-6: Interleukin-6
IPPV: Invasive positive pressure ventilation
LACI: Lacunar infarcts
LDL: Low density lipoprotein
MRI: Magnetic resonance imaging
OCSP: Oxfordshire Community Stroke Project
PACI: Partial anterior circulation infarcts
PCT: Procalcitonin
POCI: Posterior circulation infarcts
TACI: Total anterior circulation infarcts
TNF-*α*: Tumor necrosis factor-alpha
VAP: Ventilator associated pneumonia
WBC: White blood cell.
